# Trends and Knowledge Gaps in the Study of Nature-Based Participation by Latinos in the United States

**DOI:** 10.3390/ijerph15061287

**Published:** 2018-06-19

**Authors:** Pooja S. Tandon, Lauren M. Kuehne, Julian D. Olden

**Affiliations:** 1 Center for Child Health, Behavior, and Development, Seattle Children’s Research Institute, Seattle, WA 98121, USA; 2 Department of Pediatrics, University of Washington, Seattle, WA 98195, USA; 3 School of Aquatic and Fishery Sciences, University of Washington, Seattle, WA 98195, USA; lkuehne@uw.edu (L.M.K.); olden@uw.edu (J.D.O.); 4 Center for Creative Conservation, University of Washington, Seattle, WA 98195, USA

**Keywords:** nature, greenspace, recreation, outdoors, health

## Abstract

Mounting evidence supports health and well-being benefits associated with nature experiences, while also highlighting race- and class-based inequalities in access and exposure. We synthesized the literature on nature contact by Latinos in the United States to assess the state of knowledge and strategically identify research needs to improve outcomes and reduce health disparities for this rapidly growing ethnic group. Our systematic review revealed 108 articles with a notable increase in number of papers over the past 3 decades. We noted that the body of research is focused on certain demographic targets (adults in urban areas) with a relative dearth of knowledge for others (children, seniors, and rural areas). Our analysis also revealed strong compartmentalizing of studies into research “clusters” based on nonoverlapping topics and types of outcomes that are measured. Although one-third of studies explored health outcomes, these studies rarely examined other outcomes or research topics. Moreover, less than 7% of studies reported on interventions. Given the potential for nature contact to enhance health and well-being, there is substantial need for multidisciplinary research that explores interactions between social, cultural, and economic factors, and how those ultimately relate to nature contact and outcomes for Latinos in the United States.

## 1. Introduction

Recent decades have witnessed widespread recognition that exposure to nature can lead to measurable psychological and physiological health benefits, as well as numerous other positive outcomes. Considerable research points to a broad range of benefits related to nature contact, including those with significant public health implications such as increased physical activity, decreased obesity, reduced stress, and improved mental health [1,2]. The direct experiences of nature also form a foundation for a sense of stewardship and active care for the environment [3]. Scientific consensus has established that protecting and restoring access to nature in different spheres of people’s lives and across all ages, social groups, and abilities can help alleviate some of the most important challenges in public health, including obesity, stress, and mental health issues [4]. 

Despite the numerous health benefits that stem from experiencing nature, there exists significant race- and class-based inequalities with respect to access, thus depriving many people of the positive benefits associated with natural light, green views, natural landscapes, and gardens and parks near their homes, schools, and workplaces [5]. Indeed, low-income and ethnic communities in the United States often lack safe, well-landscaped, and well-maintained neighborhood parks [6]. Schools in low-income minority neighborhoods are less likely to have school gardens, and in particular, large, well-resourced gardens, compared to schools in high-income neighborhoods [7]. In places where people have fewer resources, there is limited access to safe and open green spaces where people can walk, jog, or play with children, and thus fewer opportunities to meet daily recommended levels of physical activity [8]. These types of inequities are critical given some evidence that contact with nature and greenspace may disproportionately benefit disadvantaged populations, attenuating the toxic effects of poverty and reducing health disparities—the so-called “equigenic” effect [9,10]. For this reason, ensuring access to nature contact across socioeconomic groups continues to be paramount to reduce health inequalities, increase longevity, and improve health behaviors [11]. 

People of Hispanic or Latino origin are the largest ethnic minority in the United States, comprising 18% of the population (57.5 million) in 2016, and forecasted to increase to 29% (119 million) by 2060 [12]. Latinos continue to face considerable disparities with respect to lower socioeconomic standing and poor health outcomes. This ethnic group is disproportionately affected by poor conditions of daily life and shaped by structural and social position factors such as macroeconomics, cultural values, income, education, occupation, and social support systems [13]. Currently, 9 out of every 10 Latinos in the United States reside in urban areas [14], and it is well recognized that access to public green spaces and other natural assets in built environments is unequally distributed among individuals according to social position [15]. Research over the past few decades has explored topics such as preferences, practices, and access to recreation opportunities by Latinos [16,17], however, a synthesis of research focused on nature-contact is lacking.

Given the demographic significance of Latinos in the United States, the known disparities in nature access that exist for this ethnic group, and the resulting social and environmental justice implications, we sought to synthesize the existing literature on Latinos and nature contact. The specific objectives of this systematic review were to (1) quantify trends and characteristics of the extant research on outdoor/nature-based activities by Latinos in the United States; (2) identify emerging themes and bodies of knowledge; and (3) elucidate critical research gaps and make recommendations for the most promising research frontiers in support of improving outcomes and reducing health disparities for this rapidly growing ethnic group.

## 2. Materials and Methods

We conducted a systematic search following the PRISMA guidelines to identify literature relating to interactions of U.S. Latino/Latina populations with outdoor or nature-based activities. Using protocols outlined for systematic literature review [18], combinations of the following search terms were used to identify primary source publications: Latino, Hispanic, ethnicity, race, recreation, participation, access to nature, nature-based, outdoor(s), play, environmental equity, youth, child(ren), adolescent, green space, barriers, nature quality, safety, health, physical activity, leisure, and mental health. Searches were conducted in Google Scholar, Web of Science, ScienceDirect, JSTOR, PubMed, and PsychInfo databases (1900–2016), with additional articles identified in publication references; potential articles were selected based on the title and abstract content, resulting in an initial list of 159 documents. These documents were evaluated closely against criteria for inclusion, which were articles that reported primary research data relating to nature-based experiences of Latino groups. On inspection, 51 articles in the initial document list did not meet these criteria; common reasons for exclusion were a focus on general activity (vs. nature-based or outdoor activity), studies where results for Latino groups were not reported separately, or the article was a review or summary that did not present primary research data. L.M.K. conducted the searches and initial review of the articles. P.S.T. and J.D.O. validated the reviews and any discrepancies were discussed among all three authors and resolved. 

To enable a quantitative summary of research trends, each of the remaining 108 publications was assessed against a set of attributes to identify status, trends, and knowledge gaps in the literature. These attributes included basic characteristics of study year, geographic location, if the study constituted a multiethnic comparison or was exclusively Latino-focused, and the Latino group or subgroup (i.e., ancestry or country of origin) being considered. To evaluate the representation of different research approaches, each study was classified as ‘cross-sectional’, ‘controlled experiment’, ‘longitudinal study’, or ‘descriptive’ (i.e., qualitative); a very small number that did not fit into any of these approaches were classified as ‘other’. We also categorized studies according to participant demographics of age group, individual vs. family-based, survey approach, and degree of urbanization of the research setting. For studies that evaluated use of a park or parks, the scale of the park (e.g., city, national) was categorized. Lastly, we evaluated studies for their contribution across 21 potential research topics which fell into four broad categories of Study Theme, Use & Activities, Outcomes, and Barriers & Access (Table 1). Since the focus of this review was on research trends and not on specific outcomes, we did not systematically assess for bias within individual studies.

The representation of studies across attributes was summarized to evaluate research trends with respect to time, geographic areas, participant demographics, research topics, and categories. We then applied a multivariate cluster analysis to the matrix of 21 research topics using simple matching as the dissimilarity matrix and the UPGMA (Unweighted Pair Group Method with Arithmetic Mean) algorithm. The goal of cluster analysis was to reveal groups of objects within a dataset by minimizing within-group variation and maximizing between-group variation. The resulting dendrogram graphically depicts the distance at which clusters fuse or split, allowing identification of groups of studies (and strong divisions) that share similarities in the research topics explored. The cluster analysis was conducted using the vegan package in the R Statistical Computing Project [19].

## 3. Results

**Figure 1:**
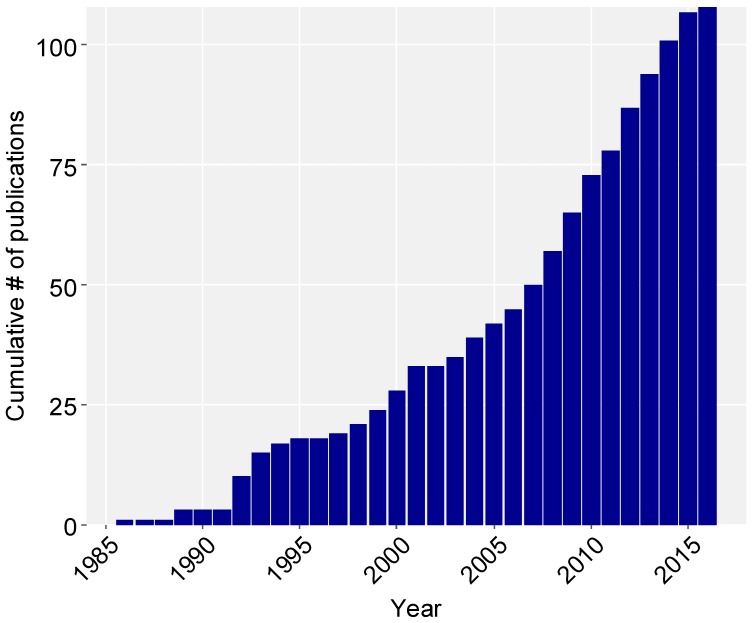
Cumulative studies (*n* = 108) by publication year that relate to participation in nature-based activities by Latino communities. Studies include those that focused solely on Latino populations as well as cross-ethnic comparisons that included Latinos.

Our systematic review revealed 108 articles or reports relating to nature experience of Latinos in the United States (Figure 1); of these, most (83%) were peer-reviewed, with the remainder divided between technical reports (12%) and grey literature (5%). A large majority of studies were cross-sectional (75%) or descriptive (16%), while controlled experiments (4%) and longitudinal (1%) studies were rare; a small number of publications (5%) were not readily classified using the other approaches. A very small number of studies considered specific Latino subgroups, with 93 studies (86%) analyzing and reporting results based on general categories of ‘Latino’, ‘Hispanic’, or ‘Latino or Hispanic’; two studies allowed respondents to identify as either ‘Hispanic’ or ‘Central American’. Studies that focused solely on a specific subgroup included Mexican Americans (9 studies), Cuban Americans (2), and Puerto Ricans (2).

**Figure 2:**
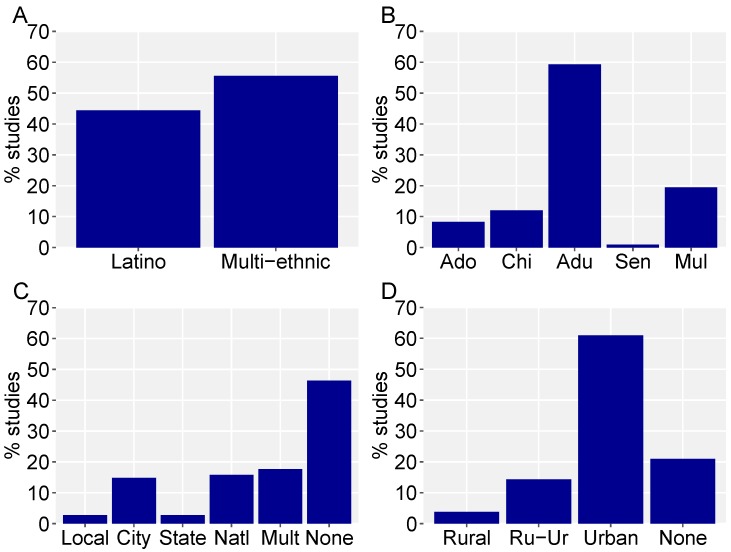
Comparative percentages of studies that focused solely on (**A**) Latino populations versus multiethnic comparisons; (**B**) Adolescents, Children, Adults, Seniors, or Multiple (referring to two groups or no specific age group); (**C**) park type in those cases when parks were a focus of the study (55% of all studies); and (**D**) urbanization of the research setting.

There has been a dramatic increase in the number of studies published in the last 20 years, with more than half of the total conducted in the last decade. Summaries of representation across demographic research targets indicated some distinct patterns of research emphasis. The majority (56%) of studies sought to contrast multiple ethnic groups, with the remainder of studies focusing solely on Latino populations (Figure 2A). Nearly 60% of studies focus on nature or outdoor activities of adults (Figure 2B). Parks were often the setting of interest (55% of studies), with study effort relatively balanced across different park types and scales (Figure 2C). Just over 60% of studies are conducted in exclusively urban settings (Figure 2D), with over half (51%) taking place in one of three U.S. states: California, Illinois, and Texas.

**Figure 3:**
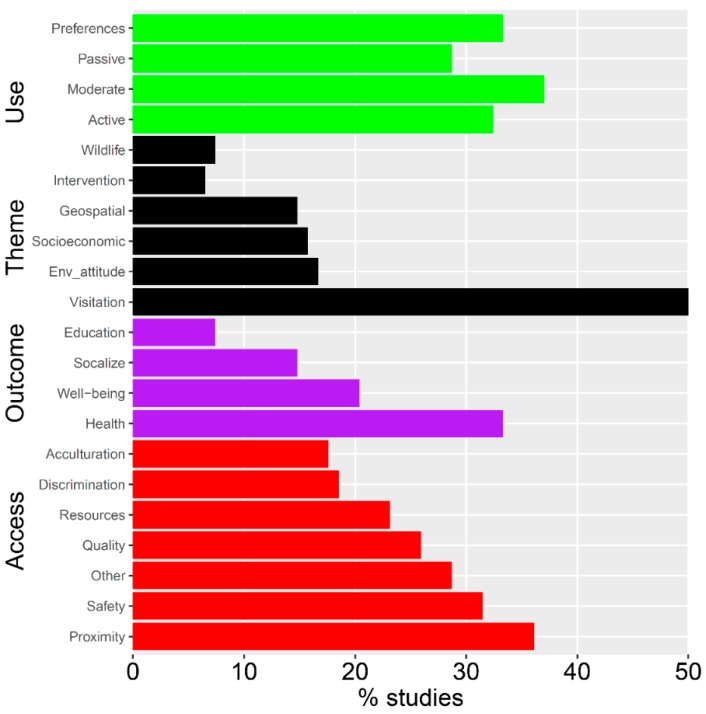
Representation of research topics across all 108 studies, grouped by broad categories (color groups). The broad categories are Use (green bars) that describe how visitors or participants used park or outdoor resources, Theme (black) that represent the general approach of the study, Outcomes (purple) that reflect measured responses or outcomes to participation in outdoor or nature-based activities, and Access (red) that depict barriers to participation or use.

Examination of representation across the 21 different research topics revealed that visitation and use has been investigated most frequently, with 50% of studies incorporating this theme (Figure 3). When outcomes of nature contact are measured, these are most commonly related to physical health (33%), in contrast to well-being, socialization, or education outcomes (>21%). Studies that have examined barriers to access in nature participation have tended to emphasize proximity (36%), while discrimination and acculturation are investigated least often (less than 20% of studies).

**Figure 4:**
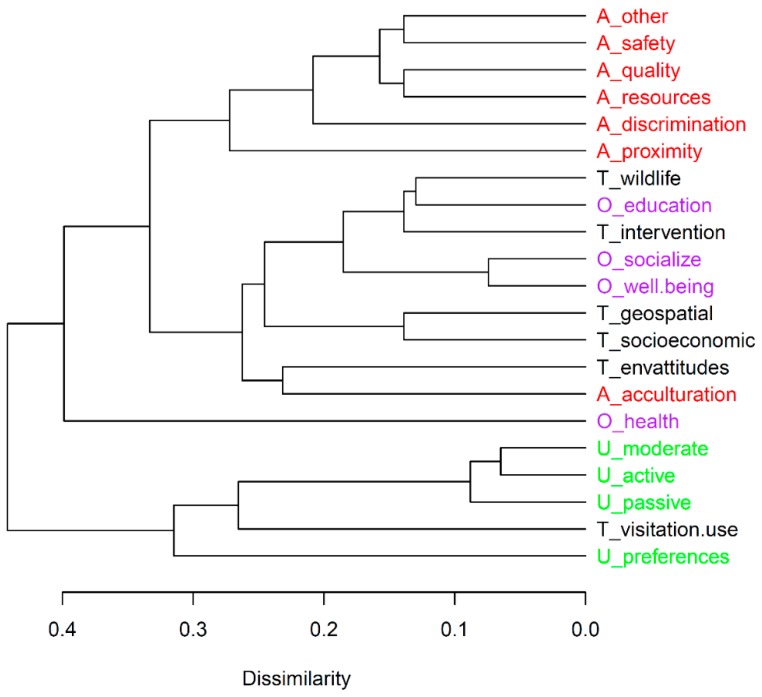
Hierarchical clustering of research topics according to published studies. Research topic labels are prefixed with the initial of the category (T = Theme, U = Use, O = Outcomes, A = Access) to which it belongs. Four clusters of research topics emerge by applying a phenon line at a dissimilarity value of 0.32.

Cluster analysis revealed four groups or clusters of research topics that are commonly examined together in published studies (Figure 4). The top of the dendrogram distinguishes studies that measured health outcomes; these publications are highly differentiated from the remaining literature and rarely incorporated other responses or study themes. The second cluster indicates a category of research focused on visitation and use as a theme, and which largely measures those responses in isolation from other themes or outcomes. The third cluster reflects a focus on barriers to access, with the different barriers to nature participation often included in the same study; the exception to this is the barrier of acculturation, which appears as part of the fourth and final cluster. The fourth cluster incorporates multiple (diverse) themes, including environmental attitudes and geospatial, socioeconomic, and intervention studies; this research cluster is where measurement of outcomes not related to health (i.e., education, socialization, and well-being) tends to occur. 

## 4. Discussion

Evidence continues to mount for the role of place in shaping health outcomes [20]. As succinctly stated by the Robert Wood Johnson Foundation Commission to Build a Healthier America, “people can make healthier choices if they live in neighborhoods that are safe, free from violence, and designed to promote health” [21]. Our systematic review revealed a notable increase in the number of papers examining aspects of nature contact within Latino populations of the United States over the past three decades. Despite this favorable trend, our review also demonstrates that the body of research (and state of knowledge) is currently dominated by a small number of research themes and topics, and individual studies have tended to be overwhelmingly narrow in their focus. 

We found that previous research efforts have predominantly focused on the context of Visitation & Use, which represented half of all studies available. These types of studies often take place in parks of varying spatial scales and are designed around understanding how to increase participation across multiple ethnic groups. As a result, although important insights are gained into park usage, activities, aesthetic preferences, and (sometimes) barriers to access, these insights are often location-specific, and typically do not assess broader historical, cultural, or socioeconomic contexts underlying observed patterns of participation, use, and benefits [22,23]. 

In addition to the emphasis on Visitation & Use, we also identified a strong compartmentalizing into research “clusters” based on the topics and types of outcomes that are measured. Visitation & Use studies typically focused on activities, while Barriers & Access tended to be studied in isolation from other topics and outcomes. Notably, although one-third of studies explored health outcomes, these studies only rarely examined other outcomes or research topics. Given the challenges inherent in multi- or interdisciplinary work, these trends may be difficult to overcome. However, the ability to unravel the complex and diverse issues that dictate nature experiences (and corresponding health and well-being outcomes) for Latino populations is likely to require collaborations across the disciplines of health, psychology, leisure studies, and public policy. For example, two studies adopted comprehensive survey-based approaches to examine issues of access to nature-based activities, while also considering preferences and outcomes for Latino populations [24,25]. In recent years, a small number of studies have also sought to integrate geospatial or landscape-level information with recreational use or health and well-being outcomes [26,27]. Our review suggests a substantial need for adopting these and other integrative approaches that can identify interactions between social, cultural, and economic factors, and how those ultimately relate to nature contact and outcomes for Latinos in the United States [8,28]. 

We revealed that barriers to access and participation are investigated with promising frequency in the literature, although attention is uneven across the different types of barriers. Proximity—meaning distance required to access a location or event—has been examined in more than one-third of studies, many of which have established that Latino populations (along with other minority groups) tend to have reduced access to nature resources [29,30]. This basic restriction in access [23] is further compounded by a suite of other barriers that our review revealed were less frequently investigated. In particular, research gaps were apparent in the topics of discrimination and acculturation [31], which were studied least frequently. Given the long history of segregation and discrimination against minorities in the United States, often actively played out in the development and maintenance of public parks and spaces [22,23], the impact of various forms of discrimination on nature contact and outcomes for Latinos warrants greater attention. 

Understanding the ways in which Latino cultural values and worldview may lead to differing needs and preferences is important in improving nature participation and access. For example, Latinos tend to have large family networks that are important sources of social support (i.e., the concept of familismo) and may influence recreation practices. Some studies have also reported that pro-environmental attitudes among Latino immigrants were negatively related to acculturation, suggesting cultural differences in environmental attitudes [32]. Programs that acknowledge these types of cultural distinctions can adapt policies to create more inclusive public spaces and nature-based experiences [24]. 

A burgeoning body of evidence suggests that access to nature and green space provides children with a myriad of cognitive, emotional, and physical benefits, such as increased ability to concentrate, improved academic performance, reduced stress and aggression levels, and reduced risk of obesity [1,2,33]. However, our review revealed a relative scarcity of research examining nature exposure, health, and other well-being outcomes for Latino children and youth. Given evidence for the benefits of nature exposure at early ages and the potential for disproportionate benefits to be realized by economically disadvantaged groups (i.e., the “equigenic” effect), we recommend increased urgency for research that examines how Latino children and youth may be marginalized from daily experiences in nature.

We believe that studies that integrate health and well-being outcomes with various exposure variables have the potential to inform programs and policies that could benefit Latino populations and other underserved minorities. In this review, there were several examples of studies that examined the relationship between proximity to nature/park access and outcomes of physical activity and/or obesity [26,34,35,36,37,38,39,40,41]. In fact, the most common health outcomes studied were obesity and physical activity. Given disproportionately high rates of obesity amongst Latinos and the widespread public health implications, this focus is expected [42]; however, a broad range of other potential benefits associated with nature contact also warrant research, including mental health, sleep, blood pressure, diabetes, and birth outcomes [4,43]. More comprehensive assessment of diverse aspects of health and well-being could help bolster investment in public policies that facilitate access to nature experience, ultimately supporting population health goals [28]. 

As this review demonstrates, the vast majority of available research studies are cross-sectional; although these approaches have numerous advantages such as being able to examine numerous factors of interest and (often) being relatively low cost, ability to infer causality between exposure and outcomes is very low. More diverse and rigorous study designs including randomized controlled trials, experiments, and studies that can take advantage of natural experiments could therefore provide much-needed evidence for the benefits of nature contact/outdoor time and have greater potential to be influential. Although intervention studies were limited (<7% of all studies), several examples included in this review provided compelling evidence of ways wherein benefits associated with outdoor and nature-based activity can be achieved (Supplemental Table S1). Gatto and colleagues [44] conducted a garden-based nutrition education program for Latino youth and found positive impact on attitudes and preferences towards fruits and vegetables. Hawthorne and colleagues [45] evaluated a 16-week school lunch time walking program, which resulted in increased cardiovascular fitness in children. Many intervention studies, however, lacked robust measurement of exposures and/or specific outcomes.

Here we discuss some important limitations of this review. First, we recognize that Latinos in the United States are a heterogeneous group (subpopulations by country of origin, years living in the United States, “home country” nature experiences, class issues, etc.). However, as revealed in the literature review, potential differences across subgroups are only rarely examined or accounted for, which is due in part to the large number of cross-sectional studies that rely on standardized survey data. Future studies could aim for a more nuanced approach to defining the study population and examining the extent to which these differences can influence both exposures and outcomes. Second, we included studies where outdoor time was used as a proxy for nature contact; however, many urban areas, school yards, or even parks may not necessarily be rich in nature. Furthermore, even in natural environments, the attributes of the particular settings (e.g., parks vs. forests) may have different implications for experiences, programs, and policies. Most studies did not attempt to characterize the specific environmental attributes of study settings, which would be useful for future research. Third, given the range of topics and study designs, we were not able to combine data from different studies to attempt a quantitative meta-analysis. With additional research, particularly focusing on health outcomes, this may be possible in the future. 

## 5. Conclusions

Given the potential for nature contact to enhance health and well-being, we recommend that future research on nature contact in Latinos could be enhanced by a greater emphasis on multidisciplinary work, expanding focus to less frequently studied populations (e.g., children, families, and seniors), and including more rigorous measures and study designs for intervention studies. Such information can help environmental educators, urban planners, and environmental organizations create policies and take actions to foster Latinos’ connection and access to nature.

## Figures and Tables

**Table 1:** Twenty-one research topics (across four broad categories) used to classify individual studies (yes or no).

Category	Topic	Description
Theme		
	Socioeconomic	Examines the importance of socio-economic factors?
	Geospatial	Uses spatial or remotely-sensed data?
	Environmental attitudes	Environmental attitudes assessed?
	Visitation	Explores recreational visitation and use?
	Wildlife	Relates to interactions with fishing, hunting, or wildlife?
	Intervention	Recounts results from an intervention program?
Use and Activities		
	Activities: Active	Involves hiking, climbing, or horseback riding?
	Activities: Moderate	Involves walking, swimming, or fishing?
	Activities: Passive	Involves picnicking or socializing?
	Preferences	Explores types of amenities and modes of communication?
Outcomes		
	Health	Examines physical activity or physiological metrics?
	Well-being	Examines relaxation, travel, or solitude?
	Socialization	Examines family bonding or sense of community?
	Education	Examines nature, culture, or learning?
Barriers and Access		
	Acculturation	Is acculturation considered as a barrier to access?
	Quality	Is the quality of available resources (e.g., facility type, maintenance) considered as a barrier?
	Safety	Is safety (perceived or realized) considered as a barrier?
	Discrimination	Is discrimination (i.e., unequal attention from police, racist incidents) considered as a barrier?
	Resources	Is lack of resources (e.g., money, equipment, or transportation) considered as a barrier?
	Proximity	Are distance and/or proximity considered as a barrier?
	Other	Are additional factors (e.g., cultural relevance, language barriers) considered as a barrier?

## Supplementary Materials

The following are available online at http://www.mdpi.com/1660-4601/15/6/1287/s1, Table S1: Peer-reviewed and grey literature documents (*n* = 108) that met the final criteria for inclusion in this review (i.e., reported primary research data related to some aspect of nature experience for Latino populations in the United States). The positive reporting of each document on six broad research themes (Table 1) used in the analysis is shown using a “*”.
