# Supplementary material for: Trends and Knowledge Gaps in the Study of Nature-Based Participation by Latinos in the United States
Source: Int J Environ Res Public Health. 2018 Jun 19;15(6):1287. doi: 10.3390/ijerph15061287 (PMC6025174; doi:10.3390/ijerph15061287)
Supplement: Supplementary file 1 [file ijerph-15-01287-s001.pdf]

Table S1. Peer-reviewed and grey literature documents (n=108) that met the final criteria for inclusion in this review (i.e., reported primary research data related to some aspect of nature experience for Latino populations in the United States). The positive reporting of each document on six broad research themes (Table 1) used in the analysis is shown using a "*".

Citation	Truncated Title	Socioec	Visit&Use	Geospat	Env Att	Wildlife	Interve
Adams et al., 2006	*The Hispanic community and outdoo….*		*				
Alexander et al., 2013	*The association between recreatio….*						
Alhassan et al., 2007	*The effects of increasing outdoor….*						*
Arcury et al., 2017	*The association between recreatio….*						
Arredondo et al., 2013	*Advocating for Environmental Chan….*		*				*
Baas et al., 1993	*Influence of ethnicity on recreat….*		*				
Babey et al., 2008	*Physical activity among adolescen….*						
Blahna and Black, 1993	*Racism: A Concern for Recreation ….*						
Blahna, 1992	*Comparing the preferences of blac….*		*			*	
Bruyere et al., 2009	*A closer examination of barriers ….*						
Burger, 2000	*Recreation and risk around Los Al….*		*			*	
Burger, 2004	*Fish consumption advisories: know….*		*			*	
Burns et al., 2008	*Outdoor recreation and nontraditi….*						
Byrne, 2012	*When green is White: The cultural….*		*		*		
Carlson et al., 2010	*Racial/ethnic differences in perc….*		*				
Carr and Williams, 1992	*Social structural characteristics….*		*				
Carr and Williams, 1993	*Understanding the role of ethnici….*		*		*		
Casper and Harrolle, 2013	*Perceptions of Constraints to Lei….*	*					
Casper et al., 2013	*Gender Differences in Self-Report….*		*				
Cerin et al., 2016	*Places where preschoolers are (in….*		*	*			
Chase et al., 2016	*A comparison of quantitative and ….*				*	*	
Chavez and Olson, 2009	*Opinions of Latino outdoor recrea….*		*				
Chavez, 1993	*The Wildland-Urban Interface: His….*		*				
Chavez, 2008	*Connecting Latinos with nature….*				*		
Ching-hua et al., 2005	*Gender and ethnic variations in u….*		*		*		
Cordell et al., 2002	*Recreation and the Environment as….*		*		*		
Cronan et al., 2008a	*Physical Activity Patterns and Pr….*		*				
Cronan et al., 2008b	*Trail use among Latinos: recogniz….*		*				
Das et al., 2017	*Park-Use Behavior and Perceptions….*		*				
Dolash et al., 2015	*Factors That Influence Park Use a….*		*				
Duncan et al., 2013	*The Geography of Recreational Ope….*	*		*			
Dwyer and Barro, 2000	*Outdoor recreation behaviors and ….*		*				

Citation	Truncated Title	Socioec	Visit&Use	Geospat	Env Att	Wildlife	Interve
Dwyer, 1992	*Outdoor Recreation Participation:….*		*				
Dwyer, 1993	*Outdoor Recreation Participation:….*		*				
Fernandez et al., 2015	*Effects of Acculturation and Acce….*		*				
Fields et al., 2013	*Built environment associations wi….*						
Floyd and Gramann, 1993	*Effects of acculturation and stru….*		*				
Floyd and Gramann, 1995	*Perceptions of discrimination in ….*						
Floyd and Noe, 1993	*Understanding intra-ethnic enviro….*				*		
Floyd et al., 1993	*Ethnic factors and the use of pub….*	*					
Franzini et al., 2010	*Neighborhood characteristics favo….*	*		*			
García and White, 2006	*"Pushing" physical activity, and ….*						*
Garcia, 2014	*Does Living in Latino Neighborhoo….*			*			
Gatto et al., 2012	*LA Sprouts: A Garden-Based Nutrit….*						*
Gobster and Delgado, 1993	*Ethnicity and recreation use in C….*		*				
Gobster, 2002	*Managing Urban Parks for a Racial….*		*				
Gómez and Malega, 2007	*Residential attributes, park use,….*	*	*	*			
Gómez, 2002	*Puerto Ricans and Recreation Part….*		*				
Gomez-Feliciano et al., 2009	*Active Living Logan Square….*						*
Gordon, 2010	*Latino Environmental Perceptions ….*	*	*		*		
Hawthorne et al., 2011	*Grand Canyon Trekkers: School-Bas….*						*
Heynen et al., 2006	*The political ecology of uneven u….*	*		*			
Hong and Anderson, 2006	*Barriers to Participation for Lat….*	*	*				
Hospodarsky and Lee, 1994	*Ethnic use of the Tonto: geograph….*		*				
Hunt and Ditton, 2001	*Perceived Benefits of Recreationa….*					*	
Hunt and Ditton, 2002	*Freshwater Fishing Participation ….*		*			*	
Hutchison, 1987	*Ethnicity and urban recreation: W….*		*				
Irwin et al., 1990	*Mexican-American/Anglo cultural d….*		*				
Johnson et al., 2004	*Ethnic Variation in Environmental….*		*		*		
Johnson et al., 2005	*Acculturation via nature-based ou….*		*			*	
Johnson Gaither, 2015	*Smokestacks, Parkland, and Commun….*	*		*			
Johnson-Gaither, 2011	*Latino park access: Examining env….*	*		*			
Kim et al., 2014	*The Role of Landscape Spatial Pat….*			*			
Kim et al., 2016	*Urban Natural Environments, Obesi….*			*			
Larson et al., 2011	*Childrens Time Outdoors: Results ….*						
Larson et al., 2013	*Young People's Outdoor Recreation….*		*				
Larson et al., 2014	*Physical activity levels and pref….*		*				
Le, 2012	*Hispanic and white visitors in US….*		*				
Lebron et al., 2016	*A Community Needs Assessment of t….*						
Lopez et al., 2007	*Texas Latino college student atti….*				*		
Madsen et al., 2014	*Justice and immigrant Latino recr….*		*	*			

Citation	Truncated Title	Socioec	Visit&Use	Geospat	Env Att	Wildlife	Interve
McNeill et al., 2015	*Walking Trail Use among a Sample ….*		*				
Mora-Trejos, 2015	*Values Assigned to National Parks….*				*		
Noe and Snow, 1990	*Hispanic Cultural Influence on En….*				*		
Nooshin Razani et al., 2015	*Healing through Nature: A Park-Ba….*						*
O'Connor et al., 2014	*Environmental and cultural correl….*			*			
Olvera et al., 2009	*Assessing physical activity prefe….*						
Parker and Green, 2016	*A Comparative Study of Recreation….*						
Perez et al., 2015	*Neighborhood Social Cohesion and ….*						
Perez et al., 2016	*Does the social environment moder….*			*			
Perry et al., 2011a	*Rural Latino Youth Park Use: Char….*		*				
Perry et al., 2011b	*Intrapersonal, Behavioral and Env….*		*				
RAND Corporation et al., 2015	*Racial-Ethnic Variation in Park U….*		*				
Rideout and Legg, 2000	*Factors Limiting Minority Partici….*		*				
Roberts and Chitewere, 2011	*Speaking of justice: Exploring et….*						
Roberts and Rodriguez, 2008	*Use of multiple methods: An exami….*		*		*		
Roman et al., 2013	*Pathways to Outdoor Recreation, P….*						
Sallis et al., 1996	*Ethnic, socioeconomic, and sex di….*	*					
Sasidharan et al., 2005	*Cultural differences in urban rec….*	*	*				
Schultz et al., 2000	*Acculturation and Ecological Worl….*	*			*		
Shaull and Gramann, 1998	*The effect of cultural assimilati….*				*		
Shinew et al., 2013	*Crime, physical activity and outd….*						
Stanis et al., 2009	*Visitor constraints to physical a….*						
Stodolska and Shinew, 2010	*Environmental constraints on leis….*						
Stodolska et al., 2010	*Recreation participation patterns….*		*				
Stodolska et al., 2011	*Perceptions of Urban Parks as Hav….*						
Strife, 2009	*The concrete jungle: Environmenta….*				*		
Tandon et al., 2012	*Frequency of Parent-Supervised Ou….*		*				
Thapa et al., 2002	*Information Needs and Search Beha….*		*				
Tierney et al., 2001	*Cultural diversity in use of unde….*	*	*				
Tinsley et al., 2002	*Park Usage, Social Milieu, and Ps….*		*				
Umstattd Meyer et al., 2013	*Understanding contextual barriers….*						
US Army Corps, 1999	*Hispanic American Recreation at T….*		*				
Van Velsor and Nilon, 2006	*A Qualitative Investigation of th….*				*	*	
Virden and Walker, 1999	*Ethnic/Racial and Gender Variatio….*				*		
Wen and Maloney, 2011	*Latino Residential Isolation and ….*	*		*			
Wen et al., 2013	*Spatial Disparities in the Distri….*	*		*			
Wolch et al., 2005	*Parks and Park Funding in Los Ang….*	*		*			
